# Initial development of the Stress Monitoring and Response Tool (SMART): A holistic measure of stress following trauma

**DOI:** 10.1371/journal.pone.0321939

**Published:** 2025-06-02

**Authors:** Emily G. Lowery-Gionta, Haley Stritzel, Liana M. Matson, Meredith A. Bucher, Xinming An, Yinyao Ji, Qinghua Li, Samuel A. McLean, Amy B. Adler

**Affiliations:** 1 Center for Military Psychiatry and Neuroscience, Walter Reed Army Institute of Research, Silver Spring, Maryland, United States of America; 2 Institute for Trauma Recovery, Department of Psychiatry, University of North Carolina at Chapel Hill, Chapel Hill, North Carolina, United States of America; 3 Department of Anesthesiology, University of North Carolina at Chapel Hill, Chapel Hill, North Carolina, United States of America; 4 Department of Emergency Medicine, University of North Carolina at Chapel Hill, Chapel Hill, North Carolina, United States of America; University of Nicosia, CYPRUS

## Abstract

In the immediate aftermath of trauma exposure, individuals may experience an acute stress reaction (ASR). ASRs may be transient but for individuals operating in high-stakes occupations, these reactions can potentially endanger themselves and those around them. Thus, a better understanding of ASRs could facilitate development of early interventions that help prevent longer-term sequelae. Although existing measures (e.g., PCL-5, CAPS-5) target symptoms that occur in the weeks following trauma, they do not encompass the range of ASR symptoms identified in emerging research. Using data from a large-scale study conducted across emergency departments in the United States, we employed confirmatory factor analysis to identify survey items that sensitively assess ASR symptoms during the peri- and post-trauma phases. These analyses identified 23 core items that are appropriate for administration both immediately following trauma exposure and at later timepoints, as well as 11 supplementary items that can be added to the core items for assessment at later timepoints. Collectively, these items constitute the Stress Monitoring and Response Tool (SMART). Both the SMART Core Scale and the combined SMART Core Scale with Supplemental Items demonstrate good convergent and concurrent validity with several other measures of mental health, physical health, somatic symptoms, pain, and functioning. In addition, the SMART scale remains moderately-to-strongly correlated with multiple measures of symptoms and functional impairment at three months post-trauma. Consequently, the SMART can be used to assess individuals in clinical settings, predict trajectories of recovery, and inform tailoring of interventions across time. Future studies should be conducted to assess the potential utility of the SMART as a decision aid in high-intensity occupational contexts.

## Introduction

Adverse posttraumatic neuropsychiatric sequelae (APNS) following exposure to a traumatic event can disrupt normal functioning and degrade quality of life. To date, research has largely focused on those *persistent* APNS that constitute Acute Stress Disorder (ASD) or Posttraumatic Stress Disorder (PTSD), which are diagnosed when symptoms persist for at least 3 days or at least 1 month, respectively. While such research is vital to understanding long-term APNS, an emerging body of literature has examined APNS during or shortly following a traumatic event.

Acute APNS occurring during or within the first 3 days of a traumatic event may be consistent with Acute Stress Reaction (ASR). ASR is described in the 11^th^ Edition of the International Classification of Diseases (ICD-11) as a response to a traumatic event comprised of transient emotional, somatic, cognitive, or behavioral symptoms that generally resolves within “a few days.”[1] The ICD-11 suggests responses that may indicate an ASR though these symptoms are primarily psychiatric. In addition, specific criteria for an ASR are not defined. The description of ASR in the ICD-11 offers a useful foundation for understanding the experience of individuals managing acute stress. Given the absence of defined criteria, however, we turned to new studies delineating ASR symptoms to target in the development of a new measure.

Studies evaluating traumatized individuals have revealed that somatic symptoms are common after various types of traumatic events [2–5]. The Advancing Understanding of RecOvery afteR traumA (AURORA) study is a multi-site investigation of APNS designed to track the emergence and trajectory of post-trauma symptoms from the acute phase to 12 months after a traumatic event [6] Findings from the AURORA study have confirmed that an array of symptoms in the initial post-trauma period extend beyond criteria defined in ASD and PTSD [7]. These symptoms include somatic concerns such as headaches, dizziness, and pain. Both traditional psychological symptoms (i.e., intrusion, avoidance, negative cognitions and mood, arousal and reactivity) and somatic symptoms independently predict subsequent functioning [8]. Such findings make clear the utility of systematically casting a wider net when assessing APNS symptoms within the acute post-trauma window.

ASR symptoms are important to identify and assess because they may imperil effective functioning in high-risk environments. Although such risk may not be experienced by individuals who find themselves in a safe environment following a traumatic event, there are times when continuing to function during or soon after a traumatic event is of paramount importance. Occupations that require continued performance in the context or immediate aftermath of a potentially traumatic event include firefighting, policing, or military service. Indeed, a recent survey of soldiers who had experienced a combat-related event found that the prevalence of ASR may be as high as 17–23% [9]. Most of the soldiers surveyed reported that their ASR lasted longer than 5 minutes; and for 1 in 5, the ASR lasted longer than a day [9]. Although it is becoming increasingly clear that ASRs occur with considerable frequency, no timely and comprehensive measure of ASR or acute APNS symptoms currently exists [10]. Generally, existing measures query individuals about their experience with acute APNS retrospectively – an approach that is susceptible to hindsight bias. Also, this approach does not facilitate medical decision making in the near aftermath of trauma exposure. Given that the AURORA study revealed a variety of symptoms assessed in the emergency department (ED) that are predictive of subsequent maladjustment, there is potential utility in developing a measure for use during this relatively acute phase.

Existing measures also typically do not capture somatic symptoms or provide a comprehensive assessment of other ASR symptoms within one single scale [11]. For example, the Posttraumatic Stress Disorder Checklist for the DSM-5 (PCL-5) [12], Stanford Acute Stress Reaction Questionnaire (SASRQ) [10,13], the Peri-traumatic Distress Inventory (PDI) [14], and the Peri-traumatic Dissociative Experiences Questionnaire (PDEQ) [15] each assess mental health symptoms but do not assess somatic ones like headaches, dizziness, and pain.

Accordingly, we endeavored to develop a more comprehensive self-report scale, the Stress Monitoring and Response Tool (SMART), to assess peritraumatic symptoms in the emotional, somatic, cognitive, and behavioral domains. The goal was development of a scale that is not only appropriate for administration in the immediate aftermath of trauma, but that also includes supplemental items appropriate for administration in the days following trauma. This approach fills the temporal gap left by the currently- available assessment tools and facilitates future research on the prediction of ASR trajectories and the efficacy of early interventions.

## Materials and methods

### Data

Data were collected in the AURORA study, a multicenter prospective cohort study of APNS among trauma survivors [6]. All methods within the AURORA study protocol were carried out in accordance with relevant guidelines and regulations and approved by the Institutional Review Board (IRB #17–0703) at the University of North Carolina, Chapel Hill. Method details have been previously described [6]. Participants were enrolled at 30 US EDs, most of which were urban academic centers. Enrollment occurred between 9/25/2017 and 12/31/2020. All participants provided written consent to participate. ED patients were eligible for inclusion in AURORA if they were 18–75 years old, presented to a participating ED for evaluation within 72 hours of an event with the potential to cause serious or life-threatening injury, were fluent in English, and had a smartphone available to participate in app-based surveys. A total of 3,829 participants enrolled in AURORA. For the current analysis, AURORA participants were included if they were injured while operating or riding in a motor vehicle or were struck by a motor vehicle, they were not admitted to the hospital, and they were initially enrolled at the ED no later than June 30, 2021, resulting in 1,644 participants included and 2,185 participants excluded from this analysis. The sample was limited to one trauma type, those experiencing trauma from motor vehicle-related incidents. We focused our sample to those experiencing motor vehicle-related incidents because it is the most common trauma type reported in our AURORA sample and thus enables assessment of the SMART’s reliability and validity without the potential confounding influence of other variables related to trauma type. See S1 Fig for more details regarding participant inclusion in the study.

### Measures

The initial AURORA measures consisted of 176 items reflecting symptom and functioning domains associated with traumatic stress (see S1 and S2 Appendices). These measures included full scales from the scientific literature, such as the PTSD Checklist for DSM-5 (PCL-5) [12], the Insomnia Severity Index (ISI) [16], the Short Form 12 Health Survey (SF-12) [17], the Sheehan disability score (SDS) [18], the Rivermead Post-Concussion Symptoms Questionnaire (RPQ) [19], the Numeric Pain Rating (NRS) and Regional Pain Scales [20,21], as well as individual items from established scales and item banks, including anxiety, depression, sleep-related impairment, and pain interference items from the Patient-Reported Outcomes Measurement Information System (PROMIS) [22–24], the Pain Catastrophizing Scale (PCS) [25], the Pittsburgh Sleep Quality Index (PSQI) [26] and the Addendum for PTSD [27], the Clinician Administered PTSD Scale (CAPS-IV) [28], the Peritraumatic Distress Inventory (PDI) [14], the Pennebaker Inventory of Limbic Languidness (PILL) [29], the Rumination Reflection Questionnaire (RRQ) [30], the Brief Dissociative Experiences Scale-Modified (DES-B) [31,32], the Michigan Critical Events Perception Scale (MCEPS) [33], and the World Health Organization (WHO) Composite International Diagnostic Interview (CIDI) [34], and DSM-5 criteria for acute stress disorder (ASD) [35]. The timing of when these items were administered relative to the participant’s ED visit is provided in S1 Appendix. Somatic symptoms were assessed in terms of their severity using 20 items from the PILL and RPQ (e.g., headache, dizziness, nausea). Some measures (i.e., PCL-5, SF-12, NRS, SDS, PROMIS Depression Short Form 8b, somatic symptoms, and ASD criteria from the DSM-5) were also used to assess the validity of the SMART.

### Analysis plan

Items were selected for consideration from the 176 possible candidate items if they assessed some aspect of acute stress as described in the literature [36], subject matter experts deemed them relevant, and they were simple declarative statements. Fifteen domains of acute stress were selected for item inclusion: pain, depression, sleep discontinuity, nightmare, anxiety, hyperarousal, avoidance, re-experiencing, somatic symptoms, concentration/thinking/fatigue, self-regulation, rumination, emotional numbing, dissociation, and irritability/anger/aggression. Initially, items were rated using 0–4, 0–5, or 0–10 response options. For items not initially rated using 0–10 response options, responses were rescaled using the rescale() function in R to standardize response options. The rescale() function maps the minimum value to 0 and maximum value to 10 proportionally. Therefore, items initially rated using a 0–4 response scale were multiplied by 2.5, and items initially rated using a 0–5 response scale were multiplied by 2.This selection process provided the initial pool of candidate items. Further down-selection was conducted to reduce redundancy, eliminate unclear items, and ensure internal consistency. Finally, the adjusted item total-correlation, which indicates the correlation between each item and the sum of the rest of the items in the scale, was used to measure internal consistency [37]. In line with best practices [37], items were removed if the adjusted item-total correlation was smaller than 0.3, which indicates they are not strongly related to our construct of interest.

Given that some items were appropriate regardless of timing (e.g., panic) and some were only applicable if administered at least 24 hours following trauma (e.g., sleep), two sets of items were developed: core items that were relevant regardless of timing (SMART Core Scale) and additional items that captured symptoms emerging after 24 hours (SMART Supplemental Items). Separate analyses were conducted on the SMART Core Scale only, and on the combined SMART Core Scale + Supplemental Items.

Cronbach’s alpha and mean inter-item correlation (MIIC) were used to determine the reliability of the SMART Core Scale and the SMART Core + Supplemental Items. Concurrent validity of the SMART Core Scale and the SMART Core Scale + Supplemental Items was tested by comparing the total scores with the ASD total score [35] and PCL-5 [12] total score measured at 2 weeks after trauma. To examine convergent validity, correlations of the SMART Core Scale and the SMART Core with Supplemental Items with scores on the SF-12 [17], PROMIS depression [22], somatic symptom scale [29], NRS Scale [20], and SDS [18] at 2 weeks after trauma were assessed. To examine predictive validity, we re-assessed the correlations of the SMART Core Scale and the SMART Core + Supplemental Items with these same measures 3 months after trauma. This approach to convergent and predictive validity is consistent with other measurement validation studies [38–40].

Next, groupings of items within the SMART Core Scale only and the SMART Core Scale + Supplemental Items were developed by subject matter experts to identify subscales that measure specific symptom domains. Confirmatory factor analysis (CFA) was used to test how well the proposed item groupings fit the data. Model fit was evaluated using the following fit indices: the root mean-square error of approximation and its 90% confidence interval (RMSEA) [41], the Tucker-Lewis index (TLI) [42], the comparative fix index (CFI) [43], and the standardized root-mean-square residual (SRMR) [44]. Based on Hu and Bentler’s [45–46] recommendations, good model fit was indicated by CFI and TLI ≥ 0.90, RMSEA ≤ 0.06, and SRMR ≤ 0.08. All analyses were conducted using Mplus version 8 [47].

Following these analyses, question wording was modified for the final versions of the SMART Core Scale and the Supplemental Items (see S1 Appendix) so that question phrasing did not specify a post-trauma time frame. In addition, items asking “how much of a problem” an individual was having with a symptom were changed to how much an individual was “experiencing the symptom.” Items asking “how often” a symptom was experienced were also changed to “how much” a symptom was experienced. These edits were made to optimize consistency across items.

## Results

### Sample description

Baseline characteristics of the sample are presented in Table 1. The mean age of participants was 34.9 (+/- 12.6 SD) years, and 45.9% were between 18 and 30 years of age. More than half of the sample (67%) was female. The sample included individuals self-identifying as Non-Hispanic Black (52%), non-Hispanic white (31%) or Hispanic (13%). Most of the participants were unmarried (78%) and employed (68%). Most annual incomes were either less than $19,000 (28%) or between $19,000 and $35,000 (28%). In terms of educational attainment, 42% of the sample had attended some college; 38% reported an education level of high school or less.

**Table 1:** Baseline Characteristics of AURORA Sample (*N* = 1,644).

Characteristic	Mean (SD) or N (%)
Age	34.9 (12.6)
Female	1099 (67)
Race	
Non-Hispanic Black	860 (52)
Non-Hispanic White	502 (31)
Hispanic	212 (13)
Other	61 (4)
Marital Status	
Married	336 (20)
Separated/Divorced/Willowed	270 (16)
Single	1029 (63)
Income	
<=$19,000	457 (28)
$19,000-$35,000	465 (28)
$35,000-$50,000	202 (12)
$50,000-$75,000	133 (8)
$75,000-$100,000	89 (5)
>$100,000	93 (6)
Employment Status	
Employed	1115 (68)
Retired/Homemaker/Student	170 (7)
Unemployed	225 (14)
Education Status	
High school or less	620 (38)
Some college	692 (42)
College or more	332 (20)
Life Event Checklists	3.6 (3.1)
Childhood Abuse Total Score	9.5 (9.8)
Sheehan Disability Score^*^	
Disrupt Social Life	5.4 (3.3)
Disrupt Work and School	5.4 (3.5)
Disrupt Family and Home	5.2 (3.2)
SF-12 Physical Health^*^	34.8 (10.2)
SF-12 Mental Health^*^	40.4 (10.2)

* Week 2 timepoint was used for these characteristics.

### Item selection

The initial selection process yielded 43 candidate items for possible inclusion in the scale. After reviewing the content of these items, three were removed due to redundancy and six were removed due to interpretation difficulties, leaving 34 items (see S1 and S2 Tables). These remaining items had means ranging from 1.78 to 6.40, and variances ranging from 4.59 to 14.45. Table 2 lists the mean, variance, and adjusted item total correlation for each item. Among the 34 items selected, 23 could appropriately be asked at any point after trauma (Core Scale), and 11 could be asked only after at least 24 hours following the trauma (Supplemental Items) (see S1 Table and S3 Appendix).

**Table 2:** Individual Scale Item Wording and Statistics for the SMART Core Scale and Combined Scale (Core and Supplemental Items). Core Scale: Cronbach’s alpha = 0.91, mean inter-item correlation = 0.31. Combined Core Scale and Supplemental Items: Cronbach’s alpha = 0.94, mean inter-item correlation = 0.31. Adjusted item-total correlation represents the correlation between each item and the entire scale score excluding that item. ^1^Variance refers to the square of the standard deviation. The response range was 0–10. Items 1–23 comprise the Core Scale; items 24–34 are the Supplemental Items.

Subscale	Item	Original Question	Mean	Variance^1^	Adjusted Item-Total Correlation (Core Items Only)	Adjusted Item-Total Correlation (Combined)
1. Mood	1. Feel Down	Over the past 24h, how often did you feel down on yourself, no good, or worthless?	3.78	11.02	0.66	0.65
	2. Sad	Over the past 24h, how often did you feel sad, depressed, or empty?	4.81	10.21	0.70	0.70
	3. Keep Distant	Over the past 24 hours, how often did you feel distant or cut off from other people?	4.00	10.61	0.66	0.64
	4. Hard to Have Positive Feelings	Over the past 24h, how often did you have trouble experiencing positive feelings?	3.37	9.56	0.66	0.66
	5. Irritable/ Angry/Aggressive	In the past 2 weeks, how much were you bothered by irritable, having angry outbursts, or acting aggressively?	3.56	10.43	0.65	0.59
2. Somatic/Cognitive	6. Headache	Over the past 24 hours, how much of a problem have you had with headaches?	5.18	10.83	0.42	0.43
	7. Dizziness	Over the past 24 hours, how much of a problem have you had with dizziness?	2.99	9.30	0.50	0.50
	8. Fatigue	Over the past 24 hours, how much of a problem have you had with fatigue?	5.44	7.94	0.49	0.49
	9. Nausea	Over the past 24 hours, how much of a problem have you had with nausea?	2.38	8.76	0.44	0.45
	10. Concentration Difficulty	Over the past 24 hours, how much of a problem have you had concentrating?	5.01	8.67	0.60	0.59
	11. Average Pain Rating	How would you rate your pain in the last 24 hours on average?	6.40	4.59	0.35	0.37
3. Dissociation	12. MCEPS In a Dream	Your experience and your feelings during and immediately after the event that brought you to ER, how often did you feel as if you were in a dream?	3.60	14.45	0.40	0.38
	13. MCEPS Someone Else	Your experience and your feelings during and immediately after the event that brought you to ER, how often did you feel as if the events around the event were happening to someone else?	1.92	10.04	0.34	0.31
	14. MCEPS Watch self	Your experience and your feelings during and immediately after the event that brought you to ER, how often did you feel as if you were watching yourself?	2.22	11.02	0.39	0.37
	15. MCEPS Passage time	Your experience and your feelings during and immediately after the event that brought you to ER, how often did you feel like you were not experiencing the normal passage of time?	4.02	13.75	0.36	0.35
	16. Unreal	In the past 2 weeks, how often did you feel like people, objects, or the world around you seem strange or unreal?	2.18	7.47	0.56	0.57
4. Hyperarousal	17. Jumpy	Over the past 24 hours, how often did you feel jumpy or easily startled?	4.58	10.57	0.65	0.67
	18. Super-alert	Over the past 24 hours, how often were you “super-alert” or watchful, or on guard?	5.89	10.33	0.51	0.52
	19. Severe Panic	Over the past 24 hours, how often did you have severe anxiety or panic?	3.56	9.19	0.65	0.68
	20. Nervous	Over the past 24 hours, how often did you feel very nervous, worried, or anxious?	5.06	9.59	0.71	0.71
5. Self-regulation	21. Try to Control	In the past few days, how often did you try to control your thoughts and feelings?	5.76	8.08	0.59	0.60
	22. Think Calm	In the past few days, how often did you make yourself think about things in a way to make you stay calm?	5.74	8.88	0.56	0.56
	23. Notice Feelings	In the past few days, how often did you simply notice your feelings and continue with what you were doing?	5.49	6.53	0.32	0.33
6. Avoidance and Re-experiencing	24. Avoid Memory Event	Over the past 24 hours, how often did you avoid memories, thoughts, or feelings related to the event?	4.73	9.53	---	0.53
	25. Avoid External Memory	Over the past 24 hours, how often did you avoid external reminders of the event? (e.g., people, places, conversations, or activities)	4.47	10.08	---	0.66
	26. Reexperiencing Repeat Memory	Over the past 24 hours, how often did you have repeated, disturbing, and unwanted memories of the event?	5.32	10.16	---	0.47
	27. Reexperiencing Upset Event	Over the past 24 hours, how often did you feel very upset when something reminded you of the event?	5.62	9.84	---	0.67
	28. Reexperiencing – Physical Reaction	Over the past 24 hours, how often did you have strong physical reactions when something reminded you of the event, like heart pounding, trouble breathing, or sweating?	4.40	10.42	---	0.69
	29. Rehash	Over the past 24 hours, how often did you find yourself “re-hashing” the circumstances related to the event in your mind?	6.15	9.62	---	0.57
7. Insomnia	30. Problem Falling Asleep	Over the last few nights, how much of a problem have you had falling asleep?	4.93	8.72	---	0.47
	31. Problem Stay Asleep	Over the last few nights, how much of a problem have you had staying asleep all night?	5.32	8.74	---	0.48
	32. Wakeup Early	Over the last few nights, how much of a problem have you had waking up too early in the morning?	4.87	10.01	---	0.42
8. Nightmare	33. Nightmare Event	Over the last few nights, how much of a problem have you had with nightmares or bad dreams about the event?	2.93	10.16	---	0.62
	34. Panic during Sleep	Over the last few nights, how much of a problem have you had with panic attacks during the night?	1.78	6.73	---	0.59

### Scale reliability and validity

Scale reliability findings are summarized in Table 2. Cronbach’s alphas were 0.91 for the SMART Core Scale only and 0.94 for the SMART Core Scale + Supplemental Items. MIICs were 0.31 for both the Core Scale only and the Core Scale + Supplemental Items. These results indicated good scale reliability [48,49].

Concurrent validity with trauma-related constructs [50] is summarized in Table 3. At 2 weeks post-trauma, correlations between the SMART Core Scale and the ASD and PTSD scales were 0.75 (*p *< 0.001) and 0.80 (*p *< 0.001), respectively. The SMART Core Scale + Supplemental Items was also highly correlated with the ASD (*r =* 0.77, *p *< 0.001) and PTSD (*r = *0.81, *p *< 0.001) scales. These correlations indicate that both versions of the SMART adequately address several of the same domains assessed by the ASD and PTSD scales (i.e., there is good concurrent validity), without affecting the ability of the SMART to assess additional domains (e.g., somatic symptoms).

**Table 3:** Correlations between the SMART and Traditional Scales of Acute Stress and Somatic Symptoms.

Domain	Timepoint	Core Items Only	Combined Scale
**ASD** ^1^	Week 2	0.75	0.77
**PCL-5** ^2^	Week 2	0.80	0.81
	Month 3	0.61	0.61
**SF-12 MHS** ^3^	Week 2	-0.57	-0.57
	Month 3	-0.45	-0.44
**Depression** ^4^	Week 2	0.70	0.69
	Month 3	0.53	0.52
**SF-12 PHS** ^5^	Week 2	-0.17	-0.18
	Month 3	-0.23	-0.24
**Somatic Symptom Total Score** ^6^	Week 2	0.72	0.71
	Month 3	0.53	0.53
**NRS Pain Score** ^7^	Week 2	0.39	0.40
	Month 3	0.32	0.32
**SDS Total Score** ^8^	Week 2	0.47	0.47
	Month 3	0.43	0.43

*p*-values for all correlations are less than 0.001.

^1^Acute Stress Disorder (ASD) scale is only available at week 2 timepoint.

^2^Post-Traumatic Stress Disorder Checklist for DSM-5 (PCL-5)

^3^12-Item Short Form Health Survey (SF-12) Mental Health score

^4^Patient-Reported Outcomes Measurement Information System (PROMIS) Depression Short Form 8b T-score

^5^12-Item Short Form Health Survey (SF-12) Physical Health score

^6^Somatic symptom scale adapted from items in the Pennebaker Inventory of Limbic Languidness (PILL) and the Rivermead Post-Concussion Symptoms Questionnaire (RPQ)

^7^Numeric Rating Scale (NRS) Pain Score

^8^Sheehan Disability Scale (SDS) total score

Correlations between SMART Scale scores and other health-related constructs were determined at 2 weeks post-trauma to assess convergent validity (Table 3). Specifically, the SMART Core Scale scores were correlated with mental health domains (SF-12 MHS; *r = *-0.57, *p *< 0.001) and PROMIS depression (*r = *0.70, *p *< 0.001). In addition, the SMART Core Scale scores correlated with measures of physical health (SF-12 PHS; *r = *-0.17, *p *< 0.001), somatic symptoms (PILL and RPQ, *r *= 0.72, *p *< 0.001) and pain (NRS pain score; *r *= 0.39, *p *< 0.001). Furthermore, the SMART Core Scale scores correlated with functioning as measured by the Sheehan Disability Scale (*r *= 0.47, *p *< 0.001). Similar results were observed for the combined SMART Core Scale + Supplemental Items as well (Table 3). These results reveal that both versions of the SMART Scale have good convergent validity with a variety of existing measures.

In terms of predictive validity with trauma-related constructs (Table 3), the correlation between the SMART Core Scale only and PCL-5 at 3 months post-trauma was 0.61 (*p *< 0.001). The correlation between the SMART Core Scale + Supplemental Items and PCL-5 at 3 months post-trauma was also 0.61 (*p* < 0.001). In addition, SMART Core Scale scores were predictive of health-related constructs at 3 months post-trauma (Table 3), including mental health domains (SF-12 MHS; *r *= -0.45, *p *< 0.001; and PROMIS depression; *r = *0.53, *p *< 0.001). Core Scale scores also correlated with measures of physical health (SF-12 PHS; *r = *-0.23, *p *< 0.001), the sum of severity ratings for 20 somatic symptoms (e.g., headaches, dizziness, and nausea; *r *= 0.53, *p *< 0.001) and pain (NRS pain score; *r *= 0.32, *p *< 0.001). Furthermore, Core Scale scores correlated with functioning as measured by the Sheehan Disability Scale (*r *= 0.43, *p *< 0.001). Similar results were observed for the combined SMART Core Scale with Supplemental Items as well (see Table 3).

In all, these results revealed that the SMART Core Scale and the SMART Core Scale + Supplemental Items retain good predictive validity for symptoms and functional impairment for several months following trauma.

### Subscale validation

Subject matter experts in emergency medicine and psychiatric trauma identified five subscales of symptom items within the Core Scale: mood, somatic, dissociation, hyperarousal, and self-regulation. An additional three subscales (avoidance and reexperiencing, insomnia, and nightmare) were identified in the Supplemental Items, resulting in a total of eight subscales.

In a CFA model of the Core Scale only, factor loadings ranged from 0.41 to 0.88 (see Table 4). Fit statistics for this model were CFI = 0.90, TLI = 0.89, RMSEA = 0.07, and SRMR = 0.07. In the CFA model of the Core Scale + Supplemental Items, factor loadings ranged from 0.41 to 0.88 and model fit statistics were CFI = 0.91, TLI = 0.90, RMSEA = 0.05, and SRMR = 0.06. These results provide support for the construct validity of the subscales.

**Table 4:** SMART Subscale Factor Structure and Loadings from Confirmatory Factor Analysis (CFA).

Subscale	Item	Factor Loading for Core Items Only*	Factor Loading for Combined Scale**
1. Mood	1. Feel Down	0.85	0.85
	2. Sad	0.88	0.88
	3. Keep Distant	0.78	0.78
	4. Hard to Have Positive Feelings	0.82	0.83
	5. Irritable/Angry/Aggressive	0.58	0.58
2. Somatic and Cognitive	6. Headache	0.68	0.68
	7. Dizziness	0.78	0.77
	8. Fatigue	0.62	0.62
	9. Nausea	0.69	0.69
	10. Concentration Difficulty	0.71	0.71
	11. Average Pain Rating	0.44	0.45
3. Dissociation	12. MCEPS In a Dream	0.73	0.72
	13. MCEPS Someone Else	0.56	0.56
	14. MCEPS Watch Self	0.66	0.66
	15. MCEPS Passage Time	0.64	0.64
	16. Unreal	0.45	0.46
4. Hyperarousal	17. Jumpy	0.73	0.74
	18. Super-alert	0.53	0.53
	19. Severe Panic	0.76	0.78
	20. Nervous	0.83	0.81
5. Self-regulation	21. Try to Control	0.82	0.83
	22. Think Calm	0.79	0.77
	23. Notice Feelings	0.41	0.41
6. Avoidance and Re-experiencing	24. Avoid Memory Event		0.62
	25. Avoid External Memory		0.66
	26. Reexperiencing Repeat Memory		0.80
	27. Reexperiencing Upset Event		0.80
	28. Reexperiencing – Physical Reaction		0.75
	29. Rehash		0.68
7. Insomnia	30. Problem Fall Asleep		0.66
	31. Problem Stay Asleep		0.79
	32. Wakeup Early		0.63
8. Nightmare	33. Nightmare Event		0.77
	34. Panic during Sleep		0.75

* Using 23 questions and 5 subscales for SMART Core Scale only, CFI = 0.90, TLI = 0.89, RMSEA = 0.07, SRMR = 0.07.

** Using 34 questions and 8 subscales for SMART Core and Supplemental Items questions, CFI = 0.91, TLI = 0.90, RMSEA = 0.05, SRMR = 0.06.

## Discussion

The evidence that a broad range of symptoms are missed by current assessment tools during the immediate post-trauma period is compelling [2,51]. To address this gap, the present study was conducted with the goal of developing the SMART, a comprehensive measure of symptoms that can be used during an ASR.

The SMART was designed to assess a broad range of APNS. Two sets of items were identified: one set of 23 core items that were designed for the immediate post-trauma period and anytime thereafter, and one set of 11 supplemental items to be asked at least 24 hours following the traumatic event. Both scales demonstrated high internal reliability, suggesting that the items reflecting a variety of symptom domains are related to one another.

Overall, the present findings revealed that both the SMART Core Scale and combined SMART Core Scale + Supplemental Items correlated highly with existing scales measuring ASD and PTSD and moderately with a validated measure of depression, indicating that both SMART versions have good convergent validity with traditional measures of mental health symptoms. Both versions of the SMART also correlated moderately well with measures of somatic symptoms and acute pain, suggesting both versions capture additional symptom domains. Moreover, both versions of the SMART predicted persistent symptoms over time. Specifically, the SMART Core Scale and the SMART Core Scale + Supplemental Items were negatively correlated with mental and physical health and positively correlated with subsequent pain and functional impairment at 3 months post-trauma. Collectively, these results indicate that the SMART is tapping into a broad range of mental health and somatic symptoms and can be used to identify individuals at risk for difficulties over time. These findings suggest that administration of the SMART during the acute phase can help identify those individuals at greatest risk for long-term health and functioning sequelae.

The SMART contributes to the field of trauma care by enabling assessment of a range of APNS domains. Typically, existing tools designed for the acute post-trauma period assess a specific set of symptoms (e.g., dissociation and peri-traumatic distress). In contrast, the SMART addresses a broad array of symptoms, including mood, pain, dissociation, hyperarousal, self-regulation, avoidance, re-experiencing, insomnia, and nightmares. By surveying a broad range of symptoms across many domains, the SMART offers a holistic alternative for assessing acute stress. In clinical settings, the SMART could be a useful and comprehensive tool for helping both individual patients and clinicians understand the individual’s symptom profile and recovery trajectory, as well as identify targets for potential intervention based on the individual’s symptom profile.

The SMART tool also contributes to the field by enabling assessment of APNS *during* an ASR. In this way, the SMART extends the capability of existing tools that are only appropriate for retrospective assessment of APNS [12–15]. By adapting items so that they can be asked during an ASR (i.e., in the immediate aftermath of trauma) and throughout subsequent time periods, the SMART is uniquely suited to quantitative assessment of the trajectory of symptoms over time. Clinicians can use the SMART to quickly evaluate an individual’s response during and immediately following trauma. The SMART can also be used to determine the relationship between early APNS symptoms and the likelihood of subsequently developing intractable long-term trauma-related conditions.

The scope of the current effort was limited by reliance on a pre-selected set of initial measures developed from a comprehensive assessment of individuals in the aftermath of trauma exposure as part of the AURORA study. Thus, other relevant symptoms may have been missed, including some described in the ICD-11. For example, despair, overactivity, and stupor are included in the ICD-11 description but were not evaluated in the AURORA cohort. This limitation may be overcome in the future via qualitative assessment of individuals during the peritraumatic phase to ensure not only that all relevant symptoms have been identified, but that the ICD-11 description can be updated to include a more comprehensive list of symptoms.

Another limitation may be that the psychometric properties of the SMART Scales were based on a particular clinical sample. Specifically, study participants had recently experienced a motor vehicle-related traumatic event and were seeking treatment in hospital emergency departments. Therefore, it is unknown how the psychometric results are generalizable to populations that have experienced other types of trauma (e.g., physical or sexual assault), seek treatment elsewhere, or do not seek treatment at all. Likewise, the sample used in the current study may not reflect the general population because participation eligibility required having a cell phone. This requirement may have introduced bias regarding the socioeconomic status of participants. Moving forward, validation of the SMART scales should include a broader array of trauma types, settings and socioeconomic backgrounds.

Moreover, the items selected for the SMART Scales were assessed during the first two weeks after trauma in the original sample. Future research should be conducted to replicate and validate the present findings by obtaining more temporally precise measures during the peritraumatic phase. It is important to note that the original wording of some items was modified to achieve consistent wording among items included in the SMART Scales, and this re-wording could potentially change the meaning of the items. Future research should evaluate whether the modified items included in the SMART assess the same underlying constructs as the items using the original wording. Correlating self-report responses with observer ratings, as well as correlating SMART results obtained in the early aftermath of trauma with outcomes beyond 3 months, would also strengthen the validity and utility of the SMART Scales.

Despite these limitations, initial analyses suggest that the SMART Scales will be valuable for assessing APNS during and immediately following trauma exposure. In the near-term, the SMART Scales are well-suited for research purposes. In addition to expanding our understanding of ASRs, the SMART Scales may support the development of interventions that facilitate management of ASRs and that prevent subsequent development of more chronic stress-related disorders. To this end, it is anticipated that the SMART Scales will ultimately be used to inform the tailoring of early interventions to specific symptom domains, based on individualized, SMART-determined recovery trajectory predictions.

## Future direction and conclusions

Future research should further validate the SMART’s predictive validity by examining the relationship between SMART scores and long-term clinical outcomes assessed through standardized clinical interviews. In addition, we recommend that future research be used to validate the SMART in cross-cultural contexts [38,39], and examine the properties of the SMART in other medical and occupational contexts (e.g., first responders and the military) as well as in other trauma populations. With refinement and validation, the SMART may prove to be especially useful in high stakes occupations, where the need to sustain performance regardless of stress level is critical. In these occupations, core items can be used to evaluate symptoms in the immediate aftermath of trauma exposure. Rapid identification of symptoms can then facilitate early intervention and track symptom remission.

Altogether, the SMART Scales reflect progress in trauma assessment by providing a comprehensive measure of symptoms that can be used during and in the immediate aftermath of trauma exposure. Overall, the SMART Scales offer unique measures that may be used to facilitate the development of interventions to mitigate both the short- and long-term negative consequences of traumatic stress exposure.

## Supporting information

S1 Fig Flowchart of Patients Enrolled in AURORA. (DOCX)

S1 Appendix Original 176 AURORA Items Measuring Acute Stress. (DOCX)

S2 Appendix Measurement of Other Constructs Used in the Study. (DOCX)

S3 Appendix Stress Monitoring and Response Tool (SMART). (DOCX)

S1 Table Original 43 Survey Questions with Drop Rationale. (DOCX)

S2 Table Correlation Matrix of 34 Selected Scale Items. (XLSX)

S1 Data (XLSX)
