# Supplementary material for: Initial development of the Stress Monitoring and Response Tool (SMART): A holistic measure of stress following trauma
Source: PLoS One. 2025 Jun 2;20(6):e0321939. doi: 10.1371/journal.pone.0321939 (PMC12129313; doi:10.1371/journal.pone.0321939)
Supplement: S1 Fig — (DOCX) [file pone.0321939.s001.docx]

**Supplement Figure 1: Flowchart of Patients Enrolled in AURORA**

Total Approached at ED

N = 22,814

Ineligible based on medical record review (n= 5,566)

*Excluded via record review prior to approaching patient. Examples of exclusion criteria include:*

- *No traumatic event, event occurred more than 72 hours ago, doesn’t speak/read English, not alert/oriented, unable to follow protocol, impaired in ability to use smart phone (deaf, blind, physically impaired), age less than 18 or greater than 75*
- *Solid organ injury greater than AAST Grade 1, long bone fracture, requires chest tube, significant hemorrhage, likely admit > 72 hours, requires operation with anesthesia, not presented within 72 hours*
- *Pregnant/breastfeeding/severe hemorrhage, intracranial injury*

Refused after screening (n=729)

*Did not consent to participate in the study.*

Enrolled

N = 3,829

Potentially eligible after medical record review

N = 16,376

Final Approach

N = 21,942

Refused before completion of screening (n = 9,222)

*Patient refused or left during screening.*

Completed Screening

N = 7,154

Ineligible based on patient screening (n=2,596)

*Examples of exclusion criteria include:*

- *Police custody,*
- *no smartphone or smartphone < 1 year,*
- *no email, won't give SSN/TIN*
- *Other ineligible: Exceeds daily opioid, self-inflicted injury, domestic violence, occupational injury, Nickel allergy*

Eligible

N = 4,558

Enrolled less than 67 days, neither did complete week 8 survey

n = 847

N=2,946

Medical Data extraction form incomplete/unavailable

n= 36

Full Enrollment

N=3,793

Screening Error n = 12 and System Failure n = 860

Exclusionary total drop

*Became pregnant or incarcerated during tenure without knowledge when started*

n = 3

N=2,943

Non-MVC Trauma Type

n = 749

N=2,194

Did not discharge to home after ED

n = 137

Not initially enrolled at ED

n = 413

N=1,644

N=2,057

*Note.* ED = Emergency Department; AAST = American Association for the Surgery of Trauma (AAST); SSN = social security number; TIN = taxpayer identification numbers; MVC = motor vehicle collision
