# Supplementary material for: Initial development of the Stress Monitoring and Response Tool (SMART): A holistic measure of stress following trauma
Source: PLoS One. 2025 Jun 2;20(6):e0321939. doi: 10.1371/journal.pone.0321939 (PMC12129313; doi:10.1371/journal.pone.0321939)
Supplement: S1 Appendix — (DOCX) [file pone.0321939.s002.docx]

**Supplement Appendix 1. Original 176 AURORA Items Measuring Acute Stress**

**Survey Measures: Emergency Department (ED) and Week 2**

**Domain(s):** Acute/Proximal Threat (Fear)/Sympathetic Nervous System Function/Peritraumatic Distress, Dissociation/Detachment, Guilt & Shame

**Time point(s):** Emergency department, Week 2 (guilt and ashamed items only)

**Scale(s):** Peritraumatic Distress Inventory (PDI), Michigan Critical Events Perception Scale (MCEPS)

**Adaptations:** Changed response wording in PDI from to what extent an individual experienced each item and in MCEPS from how much an individual agreed with an item to how often an individual experienced each item (“none of the time”, “a little of the time”, “some of the time”, “most of the time”, “all or almost all of the time”)

**Number of items:** 15

**Citation(s):** Brunet A, Weiss DS, Metzler TJ, Best SR, Neylan TC, Rogers C, et al. The Peritraumatic Distress Inventory: a proposed measure of PTSD criterion A2. Am J Psychiatry. 2001;158(9):1480-5.

Michaels AJ, Michaels CE, Moon CH, Smith JS, Zimmerman MA, Taheri PA, et al. Posttraumatic stress disorder after injury: impact on general health outcome and early risk assessment. J Trauma. 1999;47(3):460-6; discussion 6-7.

| **The following questions are about your experience and your feelings during and immediately after the event that brought you into the ER/in the past two weeks. How often did you…** | | | | | |
| --- | --- | --- | --- | --- | --- |
|  | None of the time [0] | A little of the time [1] | Some of the time [2] | Most of the time [3] | All or almost all of the tim**e** [4] |
| 1. feel helpless (ED and Week 2) |  |  |  |  |  |
| 1. feel afraid for your safety? (ED and Week 2) |  |  |  |  |  |
| 1. feel like you were about to lose control of your emotions? (ED and Week 2) |  |  |  |  |  |
| 1. feel like you had difficulty controlling your bowel or bladder? (ED and Week 2) |  |  |  |  |  |
| 1. feel horrified by what happened? (ED and Week 2) |  |  |  |  |  |
| 1. have physical reactions like sweating, shaking, or pounding heart? (ED and Week 2) |  |  |  |  |  |
| 1. feel like you might pass out? (ED and Week 2) |  |  |  |  |  |
| 1. feel like you might die? (ED and Week 2) |  |  |  |  |  |
| 1. feel like you were not experiencing the normal passage of time? (ED and Week 2) |  |  |  |  |  |
| 1. feel like you were in a daze? (ED and Week 2) |  |  |  |  |  |
| 1. feel as if you were watching yourself? (ED and Week 2) |  |  |  |  |  |
| 1. feel as if the events around the event were happening to someone else? (ED and Week 2) |  |  |  |  |  |
| 1. feel as if you were in a dream? (ED and Week 2) |  |  |  |  |  |
| 1. feel guilty? (Week 2 only) |  |  |  |  |  |
| 1. feel ashamed? (Week 2 only) |  |  |  |  |  |

**Domain(s):** Somatic Symptoms

**Time point(s):** Emergency department, Week 2

**Scale(s):** Pennebaker Inventory of Limbic Languidness

**Adaptations:** Changed response wording from “how often” an individual experiences each item to “how much of a problem” an individual has with each item

**Number of items:** 20

**Citation(s):** Pennebaker JW, Watson D. The psychology of somatic symptoms. Current concepts of somatization: Research and clinical perspectives. Progress in psychiatry, No. 31. Arlington, VA, US: American Psychiatric Association; 1991. p. 21-35.

| **Using a 0-to-10 scale, where 0 means “no problem” and 10 means “a major problem,” how much of a problem do you have with each of the following symptoms right now/in the past two weeks? You can use any number between 0 and 10 to answer.** | |
| --- | --- |
|  | Number 0-10 |
| 1. Headaches |  |
| 1. Dizziness |  |
| 1. Nausea |  |
| 1. Noise sensitivity |  |
| 1. Light sensitivity |  |
| 1. Difficulty concentrating |  |
| 1. Taking longer to think |  |
| 1. Blurred vision |  |
| 1. Double vision |  |
| 1. Restlessness |  |
| 1. Upset stomach |  |
| 1. Persistent fatigue |  |
| 1. Sensitive or tender skin |  |
| 1. Ringing in the ears |  |
| 1. Itchy eyes or skin |  |
| 1. Racing heart |  |
| 1. Insomnia or difficulty sleeping |  |
| 1. Trembling or shaking hands |  |
| 1. Feeling faint |  |
| 1. Constipation and/or diarrhea |  |

**Domain(s):** Overall Pain/Persistent Pain/Chronic Regional & Widespread Allodynia & Hyperalgesia

**Time point(s):** Emergency department, Week 2

**Scale(s):** Numeric Pain Rating Scale, Regional Pain Scale

**Adaptations:** None

**Number of items:** 19

**Citation(s):** Farrar JT, Young JP, LaMoreaux L, Werth JL, Poole MR. Clinical importance of changes in chronic pain intensity measured on an 11-point numerical pain rating scale. Pain. 2001;94(2):149-58.

Wolfe F. Pain extent and diagnosis: development and validation of the regional pain scale in 12,799 patients with rheumatic disease. J Rheumatol. 2003;30(2):369-78.

| **Using a 0-to-10 scale, where 0 means “no pain” and 10 means “severe pain or tenderness,” what is the intensity of any physical pain you are having right now/in the past two weeks, considering any or all of your pains together? You can use any number between 0 and 10 to answer.**    _____ Number 0-10  **You mentioned having pain and tenderness right now. Using a 0-to-10 scale, where 0 means “no pain” and 10 means “severe pain or tenderness,” how would you rate the severity of your pain or tenderness in each of the following areas of your body right now/in the past two weeks?** | |
| --- | --- |
|  | Number 0-10 |
| 1. Head |  |
| 1. Neck |  |
| 1. Jaw |  |
| 1. Left shoulder |  |
| 1. Right shoulder |  |
| 1. Left upper arm, including elbow |  |
| 1. Right upper arm, including elbow |  |
| 1. Left lower arm, including wrist |  |
| 1. Right lower arm, including wrist |  |
| 1. Chest |  |
| 1. Upper back |  |
| 1. Lower back |  |
| 1. Abdomen |  |
| 1. Genital area, anus, and surrounding pelvic area |  |
| 1. Left hip or upper leg, including knee |  |
| 1. Right hip or upper leg, including knee |  |
| 1. Left lower leg, including ankle |  |
| 1. Right lower leg, including ankle |  |

**Domain(s):** Dissociation

**Time point(s):** Emergency department, Week 2

**Scale(s):** Brief Dissociative Experiences Scale - Modified

**Adaptations:** Edited original response wording slightly (original responses were, “not at all”, “once or twice”, “almost every day”, “about once a day”, and “more than once a day”) and changed reference period to 30 days

**Number of items:** 5

**Citation(s):** Carlson EB, Putnam FW. An update on the Dissociative Experiences Scale. Dissociation: Progress in the Dissociative Disorders. 1993;6(1):16-27.

| **How often during the 30 days before the event/past two weeks did you have each of the following experiences?** | | | | | |
| --- | --- | --- | --- | --- | --- |
|  | None of the time [1] | A little of the time [2] | Some of the time [3] | Most of the time [4] | All or almost all of the time [5] |
| 1. People, objects, or the world around you seemed strange or unreal (ED and Week 2) |  |  |  |  |  |
| 1. You felt as though you were looking through a fog so that people and things seemed far away or unclear (ED and Week 2) |  |  |  |  |  |
| 1. You "re-hashed" in your mind things you’ve said or done (ED only) |  |  |  |  |  |
| 1. You dwelt on or thought about things that happened to you for a really long time afterward (ED only) |  |  |  |  |  |
| 1. You played back in your mind how you acted in a past situation (ED only) |  |  |  |  |  |

**Domain(s):** Sustained Threat (Sensitization)/Emotional Numbing/PTSD Symptoms

**Time point(s):** Week 2

**Scale(s):** The Posttraumatic Stress Disorder Checklist for DSM-5 (PCL-5)

**Adaptations:** Modified response wording slightly (“some” instead of “moderately” and “a lot” instead of “quite a bit” and changed reference period to past two weeks

**Number of items:** 20

**Citation(s):** Blevins CA, Weathers FW, Davis MT, Witte TK, Domino JL. The Posttraumatic Stress Disorder Checklist for DSM-5 (PCL-5): Development and Initial Psychometric Evaluation. J Trauma Stress. 2015;28(6):489-98.

| **Highly stressful experiences can cause ongoing symptoms, thoughts, or behaviors that can last for months or years after the experiences happen. In the past two weeks, how much were you bothered by any of the following symptoms, thoughts, or behaviors caused either by the event that brought you to the ER when you enrolled in the study or by any other highly stressful experience that ever happened to you?** | | | | | |
| --- | --- | --- | --- | --- | --- |
|  | Not at all [0] | A little [1] | Some  [2] | A lot  [3] | Extremely[4] |
| 1. Repeated, disturbing, and unwanted memories either of the event that brought you to the ER when you enrolled in the study or any other highly stressful experience |  |  |  |  |  |
| 1. Repeated, disturbing dreams either of the event or any other highly stressful experience |  |  |  |  |  |
| 1. Suddenly feeling or acting as if either the event or any other highly stressful experience was actually happening again, as if you were actually back there reliving it |  |  |  |  |  |
| 1. Feeling very upset when something reminded you of the event or any other highly stressful experience |  |  |  |  |  |
| 1. Having strong physical reactions when something reminded you of the event or any other highly stressful experience, for example, heart pounding, trouble breathing, or sweating |  |  |  |  |  |
| 1. avoiding memories, thoughts, or feelings related either to the event that brought you to the ER when you enrolled in the study or any other highly stressful experience? |  |  |  |  |  |
| 1. avoiding external reminders of the event or any other highly stressful experience, for example, people, places, conversations, activities, objects, or situations? |  |  |  |  |  |
| 1. trouble remembering important parts either of the event or any other highly stressful experience? |  |  |  |  |  |
| 1. having strong negative beliefs about yourself, other people, or the world, for example, having thoughts that you are bad, that no one can be trusted, or that the world is completely dangerous? |  |  |  |  |  |
| 1. blaming yourself or someone else either for the event or any other highly stressful experience or what happened after it? |  |  |  |  |  |
| 1. having strong negative emotions like fear, horror, anger, guilt, or shame? |  |  |  |  |  |
| 1. loss of interest in activities that you used to enjoy? |  |  |  |  |  |
| 1. feeling distant or cut off from other people? |  |  |  |  |  |
| 1. trouble experiencing positive emotions, for example, being unable to feel happiness or have loving feelings for people close to you? |  |  |  |  |  |
| 1. feeling irritable, having angry outbursts, or acting aggressively? |  |  |  |  |  |
| 1. taking too many risks or doing things that could cause you harm? |  |  |  |  |  |
|  | Not at all [0] | A little  [1] | Some  [2] | A lot  [3] | Extremely  [4] |
| 1. being “superalert,” watchful, or on guard? |  |  |  |  |  |
| 1. feeling jumpy or easily startled? |  |  |  |  |  |
| 1. having difficulty concentrating? |  |  |  |  |  |
| 1. trouble falling or staying asleep? |  |  |  |  |  |

**Domain(s):** Sustained Threat (Sensitization)/Amotivation/Anhedonia/Disproportionate Sense of Loss, Anxiety/Potential Threat/Sympathetic Nervous System Function

**Time point(s):** Week 2

**Scale(s):** Patient-Reported Outcomes Measurement Information System (PROMIS) Depression Short Form 8b, Anxiety Short Form 7a, and Anxiety Bank items

**Adaptations:** Modified response wording from “never”, “rarely”, “sometimes”, “often”, “always” to “none of the time”, “a little of the time”, “some of the time”, “most of the time”, and “all or almost of the time” and changed reference period to past two weeks

**Number of items:** 13

**Citation(s):** Pilkonis PA, Choi SW, Reise SP, Stover AM, Riley WT, Cella D, et al. Item banks for measuring emotional distress from the Patient-Reported Outcomes Measurement Information System (PROMIS®): depression, anxiety, and anger. Assessment. 2011;18(3):263-83.

| **The next questions are about your feelings in the past 2 weeks. How often in those 2 weeks did you…** | | | | | |
| --- | --- | --- | --- | --- | --- |
|  | None of the time  [1] | A little of the time  [2] | Some of the time  [3] | Most of the time  [4] | All or almost all of the time  [5] |
| 1. feel that nothing was interesting? |  |  |  |  |  |
| 1. feel worthless? |  |  |  |  |  |
| 1. feel that you had nothing to look forward to? |  |  |  |  |  |
| 1. feel helpless? |  |  |  |  |  |
| 1. feel sad? |  |  |  |  |  |
| 1. feel like a failure? |  |  |  |  |  |
| 1. feel depressed? |  |  |  |  |  |
| 1. feel unhappy? |  |  |  |  |  |
| 1. feel hopeless? |  |  |  |  |  |
| 1. feel anxious? |  |  |  |  |  |
| 1. worry about things? |  |  |  |  |  |
| 1. have trouble relaxing? |  |  |  |  |  |
| 1. feel tense? |  |  |  |  |  |

**Domain(s):** Screener for Anger, Anxiety, Mania, and Panic

**Time point(s):** Week 2

**Scale(s):** The World Health Organization World Mental Health Composite International Diagnostic Interview

**Adaptations:** None

**Number of items:** 6

**Citation(s):** Kessler RC, Ustün TB. The World Mental Health (WMH) Survey Initiative Version of the World Health Organization (WHO) Composite International Diagnostic Interview (CIDI). Int J Methods Psychiatr Res. 2004;13(2):93-121.

| **How often in the past 2 weeks did you…** | | | | | |
| --- | --- | --- | --- | --- | --- |
|  | None of the time [1] | A little of the time  [2] | Some of the time  [3] | Most of the time [4] | All or almost all of the time  [5] |
| 1. feel afraid as if something awful might happen? |  |  |  |  |  |
| 1. have a sudden attack of panic or extreme fear? |  |  |  |  |  |
| 1. feel irritated, annoyed, or grouchy? |  |  |  |  |  |
| 1. feel so angry that you thought you might explode? |  |  |  |  |  |
| 1. feel much more hyper or wound up than usual? |  |  |  |  |  |
| 1. have thoughts race through your mind so fast you could hardly keep track of them? |  |  |  |  |  |

**Domain(s):** Activity/Pain-Related Disability/General Health

**Time point(s):** Week 2

**Scale(s):** 12-Item Short Form Survey (SF-12)

**Adaptations:** Changed reference period to past two weeks rather than in general

**Number of items:** 12

**Citation(s):** Ware J, Kosinski M, Keller SD. A 12-Item Short-Form Health Survey: construction of scales and preliminary tests of reliability and validity. Med Care. 1996;34(3):220-33.

| **We want to get a general overview of your health in the past 2 weeks. In those 2 weeks, would you say that your health was… - poor, fair, good, very good, or excellent?** | |
| --- | --- |
|  | Poor [5] |
|  | Fair [4] |
|  | Good [3] |
|  | Very good [2] |
|  | Excellent [1] |

| **Think about activities you might have done on a typical day in the past 2 weeks. In those 2 weeks, how much did your health limit you in…** | | | |
| --- | --- | --- | --- |
|  | Yes, limited a lot  [1] | Yes, limited a little  [2] | No, not limited at all  [3] |
| 1. moderate activities, such as moving a table, pushing a vacuum cleaner, bowling, or playing golf - were you limited a lot, a little, or not limited at all? |  |  |  |
| 1. climbing several flights of stairs? |  |  |  |

| **In the past 2 weeks, did you have any of the following problems with your work or other regular activities as a result of your physical health?** | | |
| --- | --- | --- |
|  | Yes  [1] | No  [0] |
| 1. Accomplished less than you would like - as a result of your physical health? |  |  |
| 1. Were limited in the kind of work or other activities - as a result of your physical health? |  |  |

| **In the past 2 weeks, did you have any of the following problems with your work or other regular activities as a result of any emotional problems, such as feeling depressed or anxious?** | | |
| --- | --- | --- |
|  | Yes  [1] | No  [0] |
| 1. Accomplished less than you would like - as a result of any emotional problems? |  |  |
| 1. Did work or other activities less carefully than usual - as a result of any emotional problems? |  |  |

| **In the past 2 weeks, how much did pain interfere with your normal work, including work outside the home and housework - not at all, a little bit, moderately, quite a bit, or extremely?** | |
| --- | --- |
|  | Not at all [1] |
|  | A little bit [2] |
|  | Moderately [3] |
|  | Quite a bit [4] |
|  | Extremely [5] |

| **In the past 2 weeks, how much of the time did you…** | | | | | | |
| --- | --- | --- | --- | --- | --- | --- |
|  | None of the time  [6] | A little of the time [5] | Some of the time  [4] | A good bit of the time  [3] | Most of the time [2] | All of the time [1] |
| 1. feel calm and peaceful - none of the time, a little, some, a good bit, most, or all of the time? |  |  |  |  |  |  |
| 1. have a lot of energy? |  |  |  |  |  |  |
| 1. feel down-hearted and blue? |  |  |  |  |  |  |

| **In the past 2 weeks, how much of the time did your physical health or emotional problems interfere with your social activities, like visiting friends or relatives - none of the time, a little, some, most, or all of the time?** | |
| --- | --- |
|  | None of the time [5] |
|  | A little of the time [4] |
|  | Some of the time [3] |
|  | Most of the time [2] |
|  | All of the time [1] |

**Domain(s):** Activity/Pain-Related Disability

**Time point(s):** Week 2

**Scale(s):** Sheehan Disability Scale (SDS)

**Adaptations:** Changed reference period to past two weeks rather than past week

**Number of items:** 5

**Citation(s):** Sheehan DV, Harnett-Sheehan K, Raj BA. The measurement of disability. Int Clin Psychopharmacol. 1996;11 Suppl 3:89-95.

| **Using a 0-to-10 scale, where 0 means “not at all disruptive” and 10 means “extremely disruptive,” how much have symptoms related to your physical health or emotional problems disrupted each of the following areas of your life in the past 2 weeks?** | | | | | | | | | | | |
| --- | --- | --- | --- | --- | --- | --- | --- | --- | --- | --- | --- |
|  | Not at all disruptive |  | | | | | | | Extremely disruptive | | |
|  | 0 | 1 | 2 | 3 | 4 | 5 | 6 | 7 | 8 | 9 | 10 |
| 1. Your work or school work |  |  |  |  |  |  |  |  |  |  |  |
| 1. Your family life or home responsibilities |  |  |  |  |  |  |  |  |  |  |  |
| 1. Your social life or leisure activities |  |  |  |  |  |  |  |  |  |  |  |

**In the past 2 weeks, how many days were you totally unable to work or carry out your usual activities because of problems with your physical or emotional health?**

_____ Number of days (Between 0 and 14)

**And how many days in the past 2 weeks were you able to work, but the quantity or quality of your work was reduced because of problems with your physical or emotional health?**

_____ Number of days (Between 0 and 14)

D**omain(s):** Activity/Persistent Pain/Persistent Somatic Symptoms/Pain-Related Disability/Chronic Regional & Widespread Allodynia & Hyperalgesia

**Time point(s):** Week 2

**Scale(s):** Patient Reported Outcomes Measurement Information System (PROMIS) Pain Interference Short Form 4a

**Adaptations:** Modified response wording slightly from “somewhat” to “some”, “quite a bit” to “a lot”, and “very much” to “extremely” and changed reference period to past two weeks rather than past week

**Number of items:** 4

**Citation(s):** Teresi JA, Ocepek-Welikson K, Cook KF, Kleinman M, Ramirez M, Reid MC, et al. Measurement equivalence of the Patient Reported Outcomes Measurement Information System® (PROMIS®) Pain Interference short form items: Application to ethnically diverse cancer and palliative care populations. Psychological Test and Assessment Modeling. 2016;58(2):309-52.

| **In the past 2 weeks, how much did pain interfere with…** | | | | | |
| --- | --- | --- | --- | --- | --- |
|  | Not at all  [1] | A little  [2] | Some  [3] | A lot  [4] | Extremely  [5] |
| 1. your day-to-day activities - not at all, a little, some, a lot, or extremely? |  |  |  |  |  |
| 1. work around the home? |  |  |  |  |  |
| 1. your ability to participate in social activities? |  |  |  |  |  |
| 1. your household chores? |  |  |  |  |  |

D**omain(s):** Pain Catastrophizing

**Time point(s):** Week 2

**Scale(s):** Pain Catastrophizing Scale (PCS) - Rumination Subscale

**Adaptations:** Modified response wording slightly from “not at all” to “none of the time”, “to a slight degree” to “a little of the time”, “to a moderate degree” to “some of the time”, “to a great degree” to “most of the time”, and “all the time” to “all or almost all of the time” and changed reference period to past two weeks rather than in general

**Number of items:** 2

**Citation(s):** Sullivan MJL, Bishop SR, Pivik J. The Pain Catastrophizing Scale: Development and validation. Psychological Assessment. 1995;7(4):524-32.

| **In the past 2 weeks, when you were in pain how much of the time did you find yourself…** | | | | | |
| --- | --- | --- | --- | --- | --- |
|  | None of the time  [1] | A little of the time  [2] | Some of the time  [3] | Most of the time  [4] | All or almost all of the time  [5] |
| 1. thinking about how much it hurt? |  |  |  |  |  |
| 1. thinking about how badly you wanted the pain to |  |  |  |  |  |

D**omain(s):** Sleep Loss – Sleep Duration

**Time point(s):** ED, Week 2

**Scale(s):** Pittsburgh Sleep Quality Index

**Adaptations:** Changed reference period to past two weeks or 30 days before the ER visit rather than the past month

**Number of items:** 1

**Citation(s):** Buysse DJ, Reynolds CF, Monk TH, Berman SR, Kupfer DJ. The Pittsburgh Sleep Quality Index: a new instrument for psychiatric practice and research. Psychiatry Res. 1989;28(2):193-213.

**The next questions are about your sleep. In the 30 days before the event/past 2 weeks, how many hours of sleep did you get at night, on average?**

_____ Number of hours (Between 0 and 24)

**Domain(s):** Nightmares

**Time point(s):** Week 2

**Scale(s):** Clinician-Adminstered PTSD Scale (CAPS-IV)

**Adaptations:** Modified question wording slightly from “once or twice” to “less than once a week”, “once or twice a week” to “1-2 nights a week”, “several times a week” to “3-4 nights a week”, and “daily or almost every day” to “every or nearly every night” and changed reference period to past two weeks rather than 30 days after the traumatic event

**Number of items:** 2

**Citation(s):** Blake DD, Weathers FW, Nagy LM, Kaloupek DG, Gusman FD, Charney DS, et al. The development of a Clinician-Administered PTSD Scale. J Trauma Stress. 1995;8(1):75-90.

| **In the past 2 weeks, how often did you have unpleasant dreams?** | |
| --- | --- |
|  | Never [0] |
|  | Less than once a week [1] |
|  | 1-2 nights a week [2] |
|  | 3-4 nights a week [3] |
|  | Every or nearly every night [4] |

| **How much distress or discomfort did your unpleasant dreams cause you in the past 2 weeks - none; mild distress, where you may not have woken up; moderate distress, you awoke but easily went back to sleep; severe distress, you had difficulty going back to sleep; or extreme distress, where you could not go back to sleep?** | |
| --- | --- |
|  | None [0] |
|  | Mild, you might not have woken up [1] |
|  | Moderate, you easily went back to sleep [2] |
|  | Severe, you had difficulty going back to sleep [3] |
|  | Extreme, you could not go back to sleep [4] |

D**omain(s):** Panic Attack During Sleep

**Time point(s):** Week 2

**Scale(s):** Pittsburgh Sleep Quality Index - Addendum (PSQI-A)

**Adaptations:** Modified response categories from “not during the past month” to “never”, “three or more times a week” to “3-4 nights a week” and added the category, “every or nearly every night”; changed reference period to past two weeks rather than the past 30 days

**Number of items:** 1

**Citation(s):** Germain, A., Hall, M., Krakow, B., Shear, M.K., Buysse, D.J. (2005). A brief sleep scale for Posttraumatic Stress Disorder: Pittsburgh Sleep Quality Index Addendum for PTSD. *Journal of Anxiety Disorders,* 19(2), 233-244.

| **In the past 2 weeks, how often did you awaken from sleep with severe anxiety or panic?** | |
| --- | --- |
|  | Never [0] |
|  | Less than once a week [1] |
|  | 1-2 nights a week [2] |
|  | 3-4 nights a week [3] |
|  | Every or nearly every night [4] |

D**omain(s):** Insomnia/Sleep Loss, Somnolence

**Time point(s):** Week 2

**Scale(s):** Insomnia Severity Index (ISS), Patient Reported Outcomes Measurement Information System (PROMIS) Sleep-Related Impairment Short Form 8a

**Adaptations:** Modified response categories slightly for sleep problem interference and difficulty in getting things done from "a little bit" to "a little", "quite a bit" to "a lot", and "very much" to "extremely" and changed reference period for these items to past two weeks rather than the past week

**Number of items:** 9

**Citation(s):** Bastien CH, Vallières A, Morin CM. Validation of the Insomnia Severity Index as an outcome measure for insomnia research. Sleep Med. 2001;2(4):297-307.

Buysse DJ, Yu L, Moul DE, Germain A, Stover A, Dodds NE, et al. Development and validation of patient-reported outcome measures for sleep disturbance and sleep-related impairments. Sleep. 2010;33(6):781-92.

| **In the past 2 weeks, how often did you…** | | | | | |
| --- | --- | --- | --- | --- | --- |
|  | Never  [0] | Less than once a week  [1] | 1-2 nights a week  [2] | 3-4 nights a week  [3] | Every or nearly every night  [4] |
| 1. have difficulty falling asleep? |  |  |  |  |  |
| 1. have difficulty staying asleep? |  |  |  |  |  |
| 1. wake up too early? |  |  |  |  |  |

| **In the past 2 weeks, how often was it difficult for you to stay awake during the day – never, less than once a week, 1 to 2 days a week, 3 to 4 days a week, or every or nearly every day?** | |
| --- | --- |
|  | Never [0] |
|  | Less than once a week [1] |
|  | 1-2 days a week [2] |
|  | 3-4 days a week [3] |
|  | Every or nearly every day [4] |

| **How satisfied were you with your sleep in the past 2 weeks - very satisfied, somewhat satisfied, not very satisfied, somewhat dissatisfied, or very dissatisfied?** | |
| --- | --- |
|  | Very satisfied [5] |
|  | Somewhat satisfied [4] |
|  | Not very satisfied [3] |
|  | Somewhat dissatisfied [2] |
|  | Very dissatisfied [1] |

| **In the past 2 weeks, how much did your sleep problem interfere with your daily functioning, such as daytime fatigue, your ability to function at work or perform daily chores, your concentration, memory, mood, or energy – not at all, a little, somewhat, a lot, or extremely?** | |
| --- | --- |
|  | Not at all [0] |
|  | A little [1] |
|  | Somewhat [2] |
|  | A lot [3] |
|  | Extremely [4] |

| **In the past 2 weeks, how difficult was it for you to get things done because you were sleepy?** | |
| --- | --- |
|  | Not at all [0] |
|  | A little [1] |
|  | Somewhat [2] |
|  | A lot [3] |
|  | Extremely [4] |

| **In the past 2 weeks, how noticeable to others were your difficulties in functioning due to your sleep problem – not at all noticeable, a little, somewhat, a lot, or very noticeable?** | |
| --- | --- |
|  | Not at all noticeable [0] |
|  | A little [1] |
|  | Somewhat [2] |
|  | A lot [3] |
|  | Very noticeable [4] |

| **How worried or distressed were you about your sleep problem in the past 2 weeks – not at all worried, a little, somewhat, a lot, or very worried?** | |
| --- | --- |
|  | Not at all worried [0] |
|  | A little [1] |
|  | Somewhat [2] |
|  | A lot [3] |
|  | Very worried [4] |

**Survey Measures: Flash Surveys Administered via Smartphone App**

D**omain(s):** Pain, Pain Rumination

**Time point(s):** Days 1, 9, 21, 31, 43, 53, 67 (responses from Day 1 used in scale)

**Scale(s):** Numeric Pain Rating Scale, Regional Pain Scale, Pain Catastrophizing Scale (PCS) - Rumination Subscale

**Adaptations:** Modified response wording slightly from “not at all” to “never”, “to a slight degree” to “rarely”, “to a moderate degree” to “sometimes”, “to a great degree” to “often”, and “all the time” to “very often” and changed reference period to past 24 hours rather than in general

**Number of items:** 11

**Citation(s):** Farrar JT, Young JP, LaMoreaux L, Werth JL, Poole MR. Clinical importance of changes in chronic pain intensity measured on an 11-point numerical pain rating scale. Pain. 2001;94(2):149-58.

Sullivan MJL, Bishop SR, Pivik J. The Pain Catastrophizing Scale: Development and validation. Psychological Assessment. 1995;7(4):524-32.

Wolfe F. Pain extent and diagnosis: development and validation of the regional pain scale in 12,799 patients with rheumatic disease. J Rheumatol. 2003;30(2):369-78.

|  | **No Pain** |  | | | | | | | **Severe Pain** | | |
| --- | --- | --- | --- | --- | --- | --- | --- | --- | --- | --- | --- |
|  | **0** | **1** | **2** | **3** | **4** | **5** | **6** | **7** | **8** | **9** | **10** |
| 1. How would you rate your pain in the past 24 hours at its worst? |  |  |  |  |  |  |  |  |  |  |  |
| 1. How would you rate your pain in the past 24 hours on average? |  |  |  |  |  |  |  |  |  |  |  |

| **Where did you have pain in the last 24 hours?** |
| --- |
| 1. Head |
| 1. Neck/Shoulder |
| 1. Arm(s) |
| 1. Chest |
| 1. Back |
| 1. Stomach |
| 1. Hip/leg(s) |

|  | **Never**  [0] | **Rarely**  [1] | **Sometimes** [2] | **Often**  [3] | **Very often** [4] |
| --- | --- | --- | --- | --- | --- |
| 1. When you were in pain in the past 24 hours, how much did you think about it hurting? |  |  |  |  |  |
| 1. When you were in pain in the past 24 hours, how much did you think about how badly you wanted the pain to stop? |  |  |  |  |  |

D**omain(s):** Sleep, Nightmares, Panic Attack During Sleep

**Time point(s):** Days 3, 15, 25, 35, 47, 57 (responses from Day 3 used in scale)

**Scale(s):** Insomnia Severity Index (ISS), Clinician-Adminstered PTSD Scale (CAPS-IV), Pittsburgh Sleep Quality Index - Addendum (PSQI-A)

**Adaptations:** For items from the insomnia severity index, modified response categories slightly from "a little bit" to "a little", "quite a bit" to "a lot", and "very much" to "extremely" and changed reference period to past few days rather than the past week

For items regarding nightmares and panic attacks during the night, changed question wording to "how much of a problem" these symptoms were rather than "how often" an individual had these symptoms, modified response wording from number of nights per week to “none”, “a little”, “some”, “a lot”, and “extremely”, and changed reference period to past few days rather than 30 days after the traumatic event

**Number of items:** 6

**Citation(s):** Bastien CH, Vallières A, Morin CM. Validation of the Insomnia Severity Index as an outcome measure for insomnia research. Sleep Med. 2001;2(4):297-307.

Blake DD, Weathers FW, Nagy LM, Kaloupek DG, Gusman FD, Charney DS, et al. The development of a Clinician-Administered PTSD Scale. J Trauma Stress. 1995;8(1):75-90.

Germain, A., Hall, M., Krakow, B., Shear, M.K., Buysse, D.J. (2005). A brief sleep scale for Posttraumatic Stress Disorder: Pittsburgh Sleep Quality Index Addendum for PTSD. Journal of Anxiety Disorders, 19(2), 233-244.

|  | **None**  [0] | **A little**  [1] | **Some** [2] | **A lot**  [3] | **Extremely** [4] |
| --- | --- | --- | --- | --- | --- |
| 1. Over the last few nights, how much of a problem have you had falling asleep? |  |  |  |  |  |
| 1. Over the last few nights, how much of a problem have you had staying asleep all night? |  |  |  |  |  |
| 1. Over the last few nights, how much of a problem have you had waking up too early in the morning? |  |  |  |  |  |
| 1. Over the last few nights, how much of a problem have you had with nightmares or bad dreams about the event? |  |  |  |  |  |
| 1. Over the last few nights, how much of a problem have you had with nightmares or bad dreams about other things? |  |  |  |  |  |
| 1. Over the last few nights, how much of a problem have you had with panic attacks during the night? |  |  |  |  |  |

D**omain(s):** Anxiety, Depression, Panic, Hyperarousal, Emotional Numbing

**Time point(s):** Days 5, 19, 29, 39, 51, 61 (responses from Day 5 used in scale)

**Scale(s):** PROMIS Depression Short Form 8b, PROMIS Anxiety Short Form 7a, Posttraumatic Stress Disorder Checklist for DSM-5 (PCL-5)

**Adaptations:** For PROMIS items, changed reference period to past 24 hours

For PCL-5 items, modified response wording from “not at all”, “a little bit”, “moderately”, “quite a bit”, and “extremely” to “never”, “rarely”, “sometimes”, “often”, and “very often”, and changed reference period to past 24 hours

**Number of items:** 8

**Citation(s):** Blevins CA, Weathers FW, Davis MT, Witte TK, Domino JL. The Posttraumatic Stress Disorder Checklist for DSM-5 (PCL-5): Development and Initial Psychometric Evaluation. J Trauma Stress. 2015;28(6):489-98.

Pilkonis PA, Choi SW, Reise SP, Stover AM, Riley WT, Cella D, et al. Item banks for measuring emotional distress from the Patient-Reported Outcomes Measurement Information System (PROMIS®): depression, anxiety, and anger. Assessment. 2011;18(3):263-83.

|  | **Never**  [0] | **Rarely**  [1] | **Sometimes** [2] | **Often**  [3] | **Very often** [4] |
| --- | --- | --- | --- | --- | --- |
| 1. Over the past 24 hours, how often did you have severe anxiety or panic? |  |  |  |  |  |
| 1. Over the past 24 hours, how often did you feel sad depressed, or empty? |  |  |  |  |  |
| 1. Over the past 24 hours, how often did you feel down on yourself, no good, or worthless? |  |  |  |  |  |
| 1. Over the past 24 hours, how often did you feel very nervous, worried, or anxious? |  |  |  |  |  |
| 1. Over the past 24 hours, how often were you “superalert” or watchful, or on guard? |  |  |  |  |  |
| 1. Over the past 24 hours, how often did you feel jumpy or easily startled? |  |  |  |  |  |
| 1. Over the past 24 hours, how often did you feel distant or cut off from other people? |  |  |  |  |  |
| 1. Over the past 24 hours, how often did you have trouble experiencing positive feelings? (for example, being unable to feel happiness or having loving feelings for people close to you) |  |  |  |  |  |

D**omain(s):** Avoidance, Re-Experiencing, Rumination

**Time point(s):** Days 4, 17, 27, 37, 49, 59 (responses from Day 4 used in scale)

**Scale(s):** Posttraumatic Stress Disorder Checklist for DSM-5 (PCL-5)

**Adaptations:** Modified response wording from “not at all”, “a little bit”, “moderately”, “quite a bit”, and “extremely” to “never”, “rarely”, “sometimes”, “often”, and “very often”, and changed reference period to past 24 hours

**Number of items:** 6

**Citation(s):** Blevins CA, Weathers FW, Davis MT, Witte TK, Domino JL. The Posttraumatic Stress Disorder Checklist for DSM-5 (PCL-5): Development and Initial Psychometric Evaluation. J Trauma Stress. 2015;28(6):489-98.

|  | **Never**  [0] | **Rarely**  [1] | **Sometimes** [2] | **Often**  [3] | **Very often** [4] |
| --- | --- | --- | --- | --- | --- |
| 1. Over the past 24 hours, how often did you avoid memories, thoughts, or feelings related to the event? |  |  |  |  |  |
| 1. Over the past 24 hours, how often did you avoid external reminders of the event? (e.g., people, places, conversations, or activities) |  |  |  |  |  |
| 1. Over the past 24 hours, how often did you have repeated, disturbing, and unwanted memories of the event? |  |  |  |  |  |
| 1. Over the past 24 hours, how often did you feel very upset when something reminded you of the event? |  |  |  |  |  |
| 1. Over the past 24 hours, how often did you have strong physical reactions when something reminded you of the event, like heart pounding, trouble breathing, or sweating? |  |  |  |  |  |
| 1. Over the past 24 hours, how often did you find yourself “re-hashing” the circumstances related to the event in your mind? |  |  |  |  |  |

D**omain(s):** Somatic Symptoms

**Time point(s):** Days 2, 11, 23, 33, 45, 55 (responses from Day 2 used in scale)

**Scale(s):** Rivermead Post-Concussive Questionnaire (RPQ)

**Adaptations:** Modified responses from a Likert response scale ranging from “not experienced at all” to “a severe problem” to a numeric scale from 0-10 and changed reference period to past 24 hours instead of comparing before and after a head injury or accident

**Number of items:** 6

**Citation(s):** King NS, Crawford S, Wenden FJ, Moss NE, Wade DT. The Rivermead Post Concussion Symptoms Questionnaire: a measure of symptoms commonly experienced after head injury and its reliability. J Neurol. 1995;242(9):587-92.

|  | **No Problem** |  | | | | | | | **Severe Problem** | | |
| --- | --- | --- | --- | --- | --- | --- | --- | --- | --- | --- | --- |
|  | **0** | **1** | **2** | **3** | **4** | **5** | **6** | **7** | **8** | **9** | **10** |
| 1. Over the past 24 hours, how much of a problem have you had with fatigue? |  |  |  |  |  |  |  |  |  |  |  |
| 1. Over the past 24 hours, how much of a problem have you had concentrating? |  |  |  |  |  |  |  |  |  |  |  |
| 1. Over the past 24 hours, how much of a problem have you had taking longer to thing? |  |  |  |  |  |  |  |  |  |  |  |
| 1. Over the past 24 hours, how much of a problem have you had with headaches? |  |  |  |  |  |  |  |  |  |  |  |
| 1. Over the past 24 hours, how much of a problem have you had with dizziness? |  |  |  |  |  |  |  |  |  |  |  |
| 1. Over the past 24 hours, how much of a problem have you had with nausea? |  |  |  |  |  |  |  |  |  |  |  |

D**omain(s):** Self-Regulation, Disorganization

**Time point(s):** Days 6, 41, 63 (responses from Day 6 used in scale)

**Number of items:** 5

|  | **Never**  [0] | **Rarely**  [1] | **Sometimes** [2] | **Often**  [3] | **Very often** [4] |
| --- | --- | --- | --- | --- | --- |
| 1. In the past few days, how often did you stop doing the things you wanted to do? |  |  |  |  |  |
| 1. In the past few days, how often did you try to control your thoughts and feelings? |  |  |  |  |  |
| 1. In the past few days, how often did you make yourself think about things in a way to make you stay calm? |  |  |  |  |  |
| 1. In the past few days, how often did you simply notice your feelings and continue with what you were doing? |  |  |  |  |  |
| 1. In the past few days, how often did you find it hard to communicate clearly what you wanted to say to people? |  |  |  |  |  |
