# Supplementary material for: Initial development of the Stress Monitoring and Response Tool (SMART): A holistic measure of stress following trauma
Source: PLoS One. 2025 Jun 2;20(6):e0321939. doi: 10.1371/journal.pone.0321939 (PMC12129313; doi:10.1371/journal.pone.0321939)
Supplement: S2 Appendix — (DOCX) [file pone.0321939.s003.docx]

**Supplement Appendix 2. Measurement of Other Constructs Used in the Study**

The Acute Stress Disorder (ASD) Scale, posttraumatic stress disorder (PTSD) checklist for DSM-5 (PCL-5), 12-Item Short Form Health Survey (SF-12) Mental Health and Physical Health, Numeric Rating Scale (NRS) for Pain, Sheehan Disability Score (SDS), Patient Reported Outcomes Measurement Information System (PROMIS) Depression – Short Form 8b, and a scale of post-traumatic somatic symptoms from the Post-Concussion Symptoms were used to test the validity of the scale. We used two timepoints, week 2 and month 3, for these measures. For testing the validity of our scale, we used the total scores or numeric version for all the outcomes.

**ASD Scale:** DSM-5 ASD criteria were used in AURORA. There were 14 items consisting of 5 categories: intrusion, negative mood, dissociation, avoidance, and arousal. The ASD total score ranges from 0 – 14.

**PTSD checklist for DSM-5 (PCL-5):** PCL-5 includes 20 items from 4 clusters: intrusion, avoidance, negative alterations of cognitions and mood, and alterations of arousal and reactivity. PCL-5 score ranges from 0-80.

**SF-12 Physical/Mental Health:** SF-12 is a measure of health status, with 2 summary scales and 4 health concepts in each scale. SF-12 physical/mental health score ranges from 8 – 72.

**NRS Pain Score:** In AURORA, we used NRS for pain and the range is 0 – 10.

**Sheehan disability score (SDS):** SDS is used to measure impairment in 3 domains: work, family life, and social life. Every domain range 0 – 10, and the SDS total score is 0 – 30. We used SDS total score for this study.

**Depression short form 8b:** In AURORA, we used the same scoring rules as the PROMIS Depression Short Form 8b Scale, we summed the response values and used a table to convert the raw score to T-score. The T-score ranges 37.1 – 81.1.

**Post-Traumatic Somatic Symptoms:** Post-traumatic somatic symptoms were evaluated using the Rivermead Post-Concussion Symptoms Questionnaire (RPQ) and the Pennebaker Inventory of Limbic Languidness (PILL). There were 20 items in the scale and each item ranges from 0 – 10. We used the sum score of all 20 items and the range is 0 – 200.
