# Supplementary material for: Initial development of the Stress Monitoring and Response Tool (SMART): A holistic measure of stress following trauma
Source: PLoS One. 2025 Jun 2;20(6):e0321939. doi: 10.1371/journal.pone.0321939 (PMC12129313; doi:10.1371/journal.pone.0321939)
Supplement: S3 Appendix — (DOCX) [file pone.0321939.s004.docx]

**Supplemental Appendix 3. Stress Monitoring and Response Tool (SMART)**

**Instructions:** For each question, select the number that best corresponds to what you have been experiencing.

If the event that currently bothers you the most happened within 24 hours, please answer questions 1 – 23. If the event happened more than 24 hours ago, please answer all 34 questions.

| **If less than 24 hours after the event(s)** | | | | | | | | | | | |
| --- | --- | --- | --- | --- | --- | --- | --- | --- | --- | --- | --- |
| **Rate how much you have been experiencing each of the following on a 0-10 scale, where “0” means “none” and “10” means “severe”.** | | | | | | | | | | | |
|  | None  0 | 1 | 2 | 3 | 4 | 5 | 6 | 7 | 8 | 9 | Severe  10 |
| 1. feeling down on yourself, no good, or worthless? |  |  |  |  |  |  |  |  |  |  |  |
| 2. feeling sad, depressed, or empty? |  |  |  |  |  |  |  |  |  |  |  |
| 3. feeling distant or cut off from other people? |  |  |  |  |  |  |  |  |  |  |  |
| 4. feeling trouble experiencing positive feelings? |  |  |  |  |  |  |  |  |  |  |  |
| 5. feeling irritable, having angry outbursts, or acting aggressively? |  |  |  |  |  |  |  |  |  |  |  |
| 6. headaches? |  |  |  |  |  |  |  |  |  |  |  |
| 7. dizziness? |  |  |  |  |  |  |  |  |  |  |  |
| 8. fatigue? |  |  |  |  |  |  |  |  |  |  |  |
| 9. nausea? |  |  |  |  |  |  |  |  |  |  |  |
| 10. difficulty concentrating? |  |  |  |  |  |  |  |  |  |  |  |
| 11. pain? |  |  |  |  |  |  |  |  |  |  |  |
| **Rate how much you have been experiencing each of the following on a 0-10 scale, where “0” means “none” and “10” means “a great deal”.** | | | | | | | | | | | |
|  | None  0 | 1 | 2 | 3 | 4 | 5 | 6 | 7 | 8 | 9 | A great deal  10 |
| 12. feeling as if you were in a dream? |  |  |  |  |  |  |  |  |  |  |  |
| 13. feeling that you are watching yourself? |  |  |  |  |  |  |  |  |  |  |  |
| 14. feeling that things happening to you are happening to someone else? |  |  |  |  |  |  |  |  |  |  |  |
| 15. feeling that you are not experiencing the normal passage of time? |  |  |  |  |  |  |  |  |  |  |  |
| 16. feeling that people, objects, or the world around you seem strange or unreal? |  |  |  |  |  |  |  |  |  |  |  |
| 17. feeling jumpy or easily startled? |  |  |  |  |  |  |  |  |  |  |  |
| 18. feeling “super alert” or watchful or on guard? |  |  |  |  |  |  |  |  |  |  |  |
| 19. feeling severe anxiety or panic? |  |  |  |  |  |  |  |  |  |  |  |
| 20. feeling very nervous, worried, or anxious? |  |  |  |  |  |  |  |  |  |  |  |
| 21. trying to control your thoughts and feelings? |  |  |  |  |  |  |  |  |  |  |  |
| 22. trying to make yourself think about things in a way to make you stay calm? |  |  |  |  |  |  |  |  |  |  |  |
| 23. trying to simply notice your feelings and continue with what you were doing? |  |  |  |  |  |  |  |  |  |  |  |
| **If greater than 24 hours after the event(s)** | | | | | | | | | | | |
| **Rate how much you have been experiencing each of the following on a 0-10 scale, where “0” means “none” and “10” means “a great deal”.** | | | | | | | | | | | |
|  | None  0 | 1 | 2 | 3 | 4 | 5 | 6 | 7 | 8 | 9 | A great deal  10 |
| 24. avoiding memories, thoughts, or feelings? |  |  |  |  |  |  |  |  |  |  |  |
| 25. avoiding external reminders (e.g., people, places, conversations, or activities)? |  |  |  |  |  |  |  |  |  |  |  |
| 26. having repeated, disturbing, and unwanted memories? |  |  |  |  |  |  |  |  |  |  |  |
| 27. feeling very upset when something reminded you of difficult past events? |  |  |  |  |  |  |  |  |  |  |  |
| 28. having strong reactions when something reminded you of difficult past events, like heart pounding, trouble breathing, or sweating? |  |  |  |  |  |  |  |  |  |  |  |
| 29. finding yourself “re-hashing” in your mind the circumstances related to difficult past events? |  |  |  |  |  |  |  |  |  |  |  |
| 30. having problems falling asleep? |  |  |  |  |  |  |  |  |  |  |  |
| 31. having problems staying asleep? |  |  |  |  |  |  |  |  |  |  |  |
| 32. having problems waking up too early in the morning? |  |  |  |  |  |  |  |  |  |  |  |
| 33. having nightmares or bad dreams? |  |  |  |  |  |  |  |  |  |  |  |
| 34. having panic attacks during the night? |  |  |  |  |  |  |  |  |  |  |  |
