# Supplementary material for: Initial development of the Stress Monitoring and Response Tool (SMART): A holistic measure of stress following trauma
Source: PLoS One. 2025 Jun 2;20(6):e0321939. doi: 10.1371/journal.pone.0321939 (PMC12129313; doi:10.1371/journal.pone.0321939)
Supplement: S1 Table — (DOCX) [file pone.0321939.s005.docx]

| **Supplement Table 1: Original 43 Survey Questions with Drop Rationale**  Redundant Item  Difficult to Interpret | | | |
| --- | --- | --- | --- |
| **Domain** | **Questions** | **Drop Rationale** | **Mean (SD)** |
| Pain | 1. How would you rate your pain in the last 24h on average? | With a high correlation between the two pain questions (>0.8), we risk scale redundancy. The easier to understand question (#1) was kept. | 6.40 (2.14) |
|  | 2. How would you rate your pain in the last 24h at its worst? |  | 7.29 (1.97) |
|  | 3. When you were in pain in the past 24 hours, how much did you think about it hurting? | These two questions are difficult to interpret. Level of pain intensity is likely to affect the responses to these questions. The more something hurts, the more you will want it to stop/think about it. | 7.12 (2.27) |
|  | 4. When you were in pain in the past 24 hours, how much did you think about how badly you wanted the pain to stop? |  | 7.40 (2.58) |
| Depression | 5. Over the past 24h, how often did you feel down on yourself, no good, or worthless? |  | 3.78 (3.32) |
|  | 6. Over the past 24h, how often did you feel sad, depressed, or empty? |  | 4.81 (3.20) |
|  | 7. Over the past 24h, how often did you have trouble experiencing positive feelings (e.g., being unable to feel happiness or having loving feelings for people close to you) |  | 3.38 (3.09) |
| Sleep Discontinuity | 8. Over the last few nights, how much of a problem have you had falling asleep? |  | 4.93 (2.95) |
|  | 9. Over the last few nights, how much of a problem have you had staying asleep all night? |  | 5.32 (2.96) |
|  | 10.  Over the last few nights, how much of a problem have you had waking up too early in the morning? |  | 4.87 (3.16) |
| Nightmare | 11. Over the last few nights, how much of a problem have you had with nightmares or bad dreams about the event? |  | 2.93 (3.19) |
|  | 12.  Over the last few nights, how much of a problem have you had with nightmares or bad dreams about other things? |  | 2.45 (2.92) |
|  | 13. Over the last few nights, how much of a problem have you had with panic attacks during the night? |  | 1.78 (2.60) |
| Anxiety | 14. Over the past 24 hours, how often did you have severe anxiety or panic? |  | 3.56 (3.03) |
|  | 15.  Over the past 24 hours, how often did you feel very nervous, worried, or anxious? |  | 5.06 (3.10) |
| Hyperarousal | 16. Over the past 24 hours, how often were you “super-alert” or watchful, or on guard? |  | 5.89 (3.22) |
|  | 17.  Over the past 24 hours, how often did you feel jumpy or easily startled? |  | 4.58 (3.25) |
| Avoidance | 18.  Over the past 24 hours, how often did you avoid memories, thoughts, or feelings related to the event? |  | 4.73 (3.09) |
|  | 19. Over the past 24 hours, how often did you avoid external reminders of the event? (e.g., people, places, conversations, or activities) |  | 4.47 (3.18) |
| Re-experiencing | 20.  Over the past 24 hours, how often did you have repeated, disturbing, and unwanted memories of the event? |  | 5.32 (3.19) |
|  | 21. Over the past 24 hours, how often did you feel very upset when something reminded you of the event? |  | 5.62 (3.14) |
|  | 22. Over the past 24 hours, how often did you have strong physical reactions when something reminded you of the event, like heart pounding, trouble breathing, or sweating? |  | 4.40 (3.23) |
| Somatic Symptoms | 23.  Over the past 24 hours, how much of a problem have you had with headaches? |  | 5.18 (3.29) |
|  | 24. Over the past 24 hours, how much of a problem have you had with dizziness? |  | 2.99 (3.05) |
|  | 25.  Over the past 24 hours, how much of a problem have you had with nausea? |  | 2.38 (2.96) |
| Concentration  /Thinking  /Fatigue | 26.  Over the past 24 hours, how much of a problem have you had with fatigue? |  | 5.44 (2.82) |
|  | 27. Over the past 24 hours, how much of a problem have you had concentrating? | With a high correlation between these two items (>0.8), we risk scale redundancy. The easier to understand item (#25) was kept. | 5.01 (2.94) |
|  | 28.  Over the past 24 hours, how much of a problem have you had taking longer to think? |  | 4.80 (3.03) |
| Self-Regulation | 29. In the past few days, how often did you stop doing the things you wanted to do? | This item is difficult to interpret. | 5.37 (2.99) |
|  | 30. In the past few days, how often did you try to control your thoughts and feelings? |  | 5.77 (2.84) |
|  | 31. In the past few days, how often did you make yourself think about things in a way to make you stay calm? |  | 5.74 (2.98) |
|  | 32. In the past few days, how often did you simply notice your feelings and continue with what you were doing? |  | 5.50 (2.55) |
|  | 33. In the past few days, how often did you find it hard to communicate clearly what you wanted to say to people? | This item is difficult to interpret. | 4.68 (3.22) |
| Rumination | 34. Over the past 24 hours, how often did you find yourself “re-hashing” the circumstances related to the event in your mind? - rumination |  | 6.15 (3.10) |
| Emotional Numbing | 35. Over the past 24 hours, how often did you feel distant or cut off from other people? |  | 4.00 (3.26) |
| Dissociation | 36. Your experience and your feelings during and immediately after the event that brought you to ER, how often did you feel as if you were in a dream? |  | 3.60 (3.80) |
|  | 37. Your experience and your feelings during and immediately after the event that brought you to ER, how often did you feel as if the events around the event were happening to someone else? |  | 1.92 (3.17) |
|  | 38. Your experience and your feelings during and immediately after the event that brought you to ER, how often did you feel as if you were watching yourself? |  | 2.22 (2.32) |
|  | 39. Your experience and your feelings during and immediately after the event that brought you to ER, how often did you feel like you were not experiencing the normal passage of time? |  | 4.02 (3.71) |
|  | 40. Your experience and your feelings during and immediately after the event that brought you to ER, how often did you feel like you were in a daze? | This item is difficult to interpret. | 4.70 (3.53) |
|  | 41. In the past 2 weeks, how often did you feel like people, objects, or the world around you seem strange or unreal? |  | 2.18 (2.73) |
| Irritability/Anger/Aggressive | 42. In the past 2 week, how much were you bothered by irritable, having angry outbursts, or acting aggressively? |  | 3.56 (3.22) |
| Social Withdrawal | 43. In the past 2 weeks, how much of the time did your physical health or emotional problems interfere with your social activities, like visiting friends or relatives? | This item is difficult to interpret. | 4.81 (3.16) |
| *Note.* SD = standard deviation; 24h = 24 hours; ER = emergency room | | | |
